# Familial partial lipodystrophy, Dunnigan variety - challenges for patient care during pregnancy: a case report

**DOI:** 10.1186/s13104-015-1065-4

**Published:** 2015-04-11

**Authors:** Sandra Patrícia Mota Belo, Ângela Celeste Magalhães, Paula Freitas, Davide Maurício Carvalho

**Affiliations:** Department of Endocrinology, Diabetes and Metabolism of the Centro Hospitalar de São João, Porto, Portugal; Faculty of Medicine of the University of Porto, Porto, Portugal; Instituto de Investigação e Inovação em Saúde, Universidade do Porto, Porto, Portugal

**Keywords:** Dunnigan lipodystrophy, FPLD2, Insulin resistance, Dyslipidaemia, Leptin, Pregnancy

## Abstract

**Background:**

Familial partial lipodystrophy, Dunnigan variety, is a recognised autosomal dominant disorder which is caused by heterozygous missense mutations in the lamin A/C gene. Dunnigan lipodystrophy is characterised by a variable loss of fat from the extremities and trunk, as well as an excess of subcutaneous fat in the chin and supraclavicular area. The associated metabolic abnormalities include: insulin resistance, diabetes, dyslipidaemia and low leptin levels.

**Case presentation:**

The authors studied the case of a 24-year-old caucasian pregnant woman, with a past medical history of acute pancreatitis, combined dyslipidaemia and diabetes mellitus. At 7 weeks of pregnancy she was referred to the outpatient endocrinology and obstetrics clinic for diabetes care. A physical examination revealed that she presented a loss of fat from the extremities and trunk and also had an excess of subcutaneous fat in the chin. Triglyceride levels were persistently high, and glycaemic control was only achieved through the administration of high doses of insulin (1.8 U/Kg/day). Dunnigan lipodystrophy was suspected and thus a genetic study was requested, which revealed the presence of c.1444C > T (p.Arg482Trp) heterozygote mutation in the lamin A/C gene.

**Conclusion:**

This case is used to illustrate the importance of being able to recognise the clinical signs of this rare lipodystrophic syndrome, which may cause potentially severe consequences, and also the difficulties in treating it during pregnancy.

## Background

Lipodystrophies constitute a rare, heterogeneous group of metabolic disorders, which affect adipose tissue distribution, characterised by varying degrees of body fat loss and, in some instances, an abnormal localised accumulation of subcutaneous fat [1-5]. These disorders can be divided into ‘generalised’, or ‘partial’, depending on the degree and pattern of fat loss. Moreover, the generalised and partial divisions can be further partitioned into inherited, or acquired, forms. Metabolic complications, such as hypoleptinemia, marked insulin resistance, diabetes mellitus, dyslipidaemia and hepatic steatosis, are all generally associated with this condition and their severity is related to the extent of fat loss [1,3,6].

The main subtypes of inherited lipodystrophies are autosomal recessive generalised lipodystrophy, linked to mutations of either *BSCL2* or *AGPAT2* genes [7,8], and autosomal dominant partial lipodystrophy, which is caused by mutations in the *PPARG* [9-11] or *LMNA* [12,13] genes.

The Dunnigan lipodystrophy variety, also known as familial partial lipodystrophy type 2 (FPLD2; OMIM151660), is a rare autosomal dominant disease, with an estimated prevalence of less than 1 in 10 million [3], which is caused by mutations in the *LMNA* gene, which is located in chromosome 1q21-22, encoding the nuclear proteins lamin A and C [12-14]. Lamin A and C are members of the intermediate filament protein family, and are required for nuclear lamina formation [15,16]. The mutant gene products may disrupt interaction with chromatin or other nuclear lamina proteins, resulting in abnormal differentiation and the premature death of adipocytes [17-19]. Most mutations cluster in the carboxy-terminal immunoglobulin domain of lamin A/C [3], and the majority of patients have heterozygous missense mutations, affecting codon 482 on exon 8 [20-25]. FPLD2 patients are born with normal fat distribution and after puberty they lose subcutaneous fat from their extremities, trunk and gluteal regions. Excess fat may be redistributed to the face, neck, back, labia majora and the abdominal cavity or abdominal organs, namely the liver, which accounts for the high prevalence of hepatic steatosis [26-28]. Skeletal muscle fibres in patients with FPLD2 display significant hypertrophy [29]. These patients also experience a multitude of metabolic complications, including hyperlipidaemia, hypertriglyceridaemia and diabetes. FLPD2 women, in whom the disease is usually more severe [30,31], are at high risk of having hyperandrogenism, infertility, gestational diabetes, obstetrical complications and they require extra gynaecological and obstetrical care [32].

The authors now move on to describe the case of a FPLD2 female patient who was diagnosed during pregnancy.

## Case presentation

### Prior to pregnancy

The 24-year-old caucasian female patient had a past medical history of acute pancreatitis, and experienced her first episode at the age of 20, which was complicated by superior mesenteric vein thrombosis. The second episode occurred at the age of 21. Combined dylipidemia was diagnosed in this context and was initially treated with a statin and fibrate, but without adequate control. Niacinic acid was added, with improved control. Diabetes, classified as secondary to pancreatitis, was later diagnosed at the age of 22, and was treated with insulin since the beginning of its discovery. The patient was subsequently referred to endocrinology appointments, where hypercortisolism was suspected, but excluded. She abandoned these appointments shortly after.

Her gynaecological and obstetric history was irrelevant, and she had no previous history of menstrual abnormalities, hyperandrogenism, or infertility.

### During pregnancy

**Figure 1:**
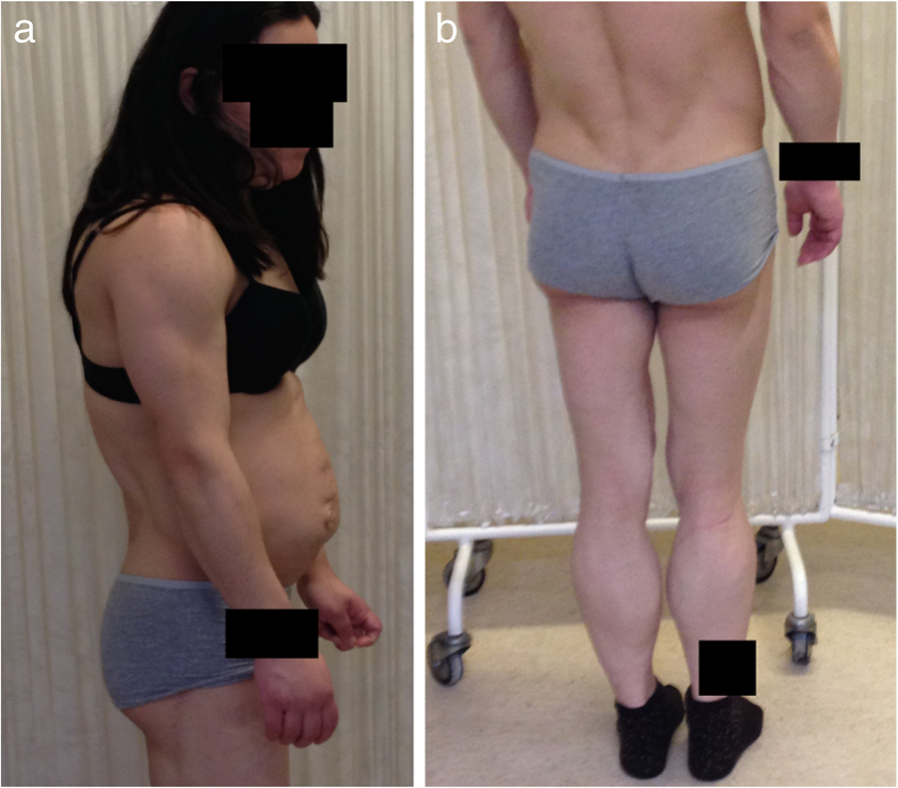
**a and b Loss of adipose tissue from the extremities and the trunk.**

**Figure 2:**
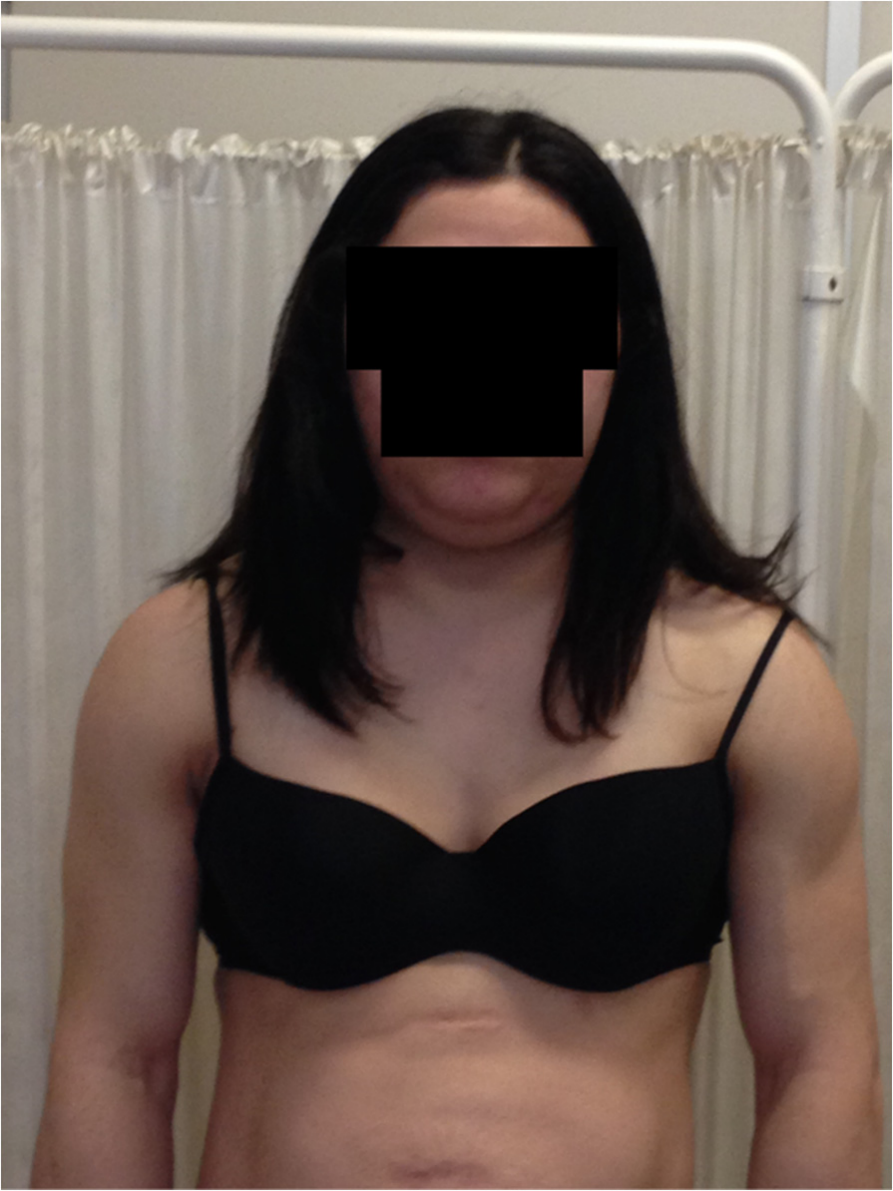
**Abnormal deposition of adipose tissue on the chin.**

At 7 weeks of pregnancy, she was referred to the outpatient endocrinology and obstetrics clinic, in the context of her diabetes. A physical examination revealed that she presented loss of fat from the extremities and trunk (Figure 1a; 1b), excess of subcutaneous fat in the chin, and apparent muscle hypertrophy (Figure 2). According to the patient, these features had been present since early adolescence. No other typical clinical features were observed during the physical examination, namely hirsutism or acanthosis nigricans. As previously noted, the therapy for diabetes was insulin, with an initial total dose of 106 U/day (1.8 U/Kg/day). The patient was also treated with heparin and salmon oil. Statin, niacinic acid and fenofibrate treatment had already been suspended at the time pregnancy was confirmed. She presented good glycaemic control (HbA1c 4.7%, without hypoglycaemia) and triglyceride levels of 1566 mg/dL (reference value <150 mg/dL), and so was prescribed treatment with gemfibrozil 600 mg, twice a day. Considering her past medical history, and the clinical and biochemical characteristics, Dunnigan lipodystrophy was suspected, and a genetic study for mutations of lamin A/C was requested. The presence of exon 8 c.1444C > T (p.Arg482Trp) heterozygote mutation in the *LMNA* gene was revealed. No other genes were sequenced.

At 12 weeks of pregnancy, the patient was admitted to the emergency department with the diagnosis of acute pancreatitis, with triglyceride levels of 9975 mg/dL. After improvement of triglyceride levels provoked by fasting, treatment began with fenofibrate 267 mg, once a day, and the patient was discharged with triglyceride levels of 655 mg/dL. One week later, on re-evaluation, she presented triglyceride levels of 2965 mg/dL. Considering the risk of a new episode of pancreatitis, the patient was then admitted for metabolic control, centred on fasting. After discharge, with triglyceride levels of 1086 mg/dL, a rigorous dietary plan was prescribed, and treatment was also started with Omacor® [highly purified omega-3 fatty acid ethyl esters (3 g tid)] and Protifar® [high protein powdered supplement (32 sp)]. Triglyceride levels remained constantly between 1000 and 2000 mg/dL until the birth. The birth was by dystocic delivery – caesarean section - because of the failure of natural labour. The operation proceeded without complications and the new-born, although macrosomic (4675 g), presented no clinical signs of having the disease, as was expected.

### After delivery

After delivery, the patient begun regular evaluation during endocrinology appointments and returned to statin (rosuvastatin 20 mg), cholestyramine (4000 mg once a day) and fibrate (fenofibrate 267 mg once a day) treatment, with suboptimal triglyceride levels control (triglyceride levels of 882 mg/dL). For diabetes treatment, metformin was added to insulin, with good glycaemic control (HbA1c < 7%).

### Family history

With regards to her family medical history, the patient has 3 healthy brothers and a healthy father. However her mother has type 2 diabetes *mellitus*, hypertriglyceridaemia and, according to the patient, she also has the same fat distribution pattern. No history of pancreatitis is known.

**Figure 3:**
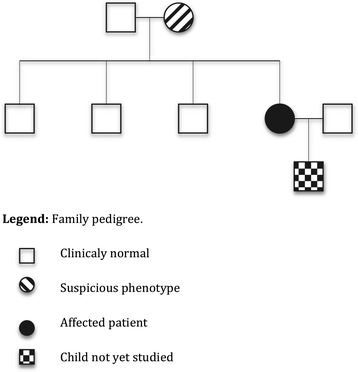
**Family pedigree.**

Medical evaluation and a genetic study, if appropriate, was offered to all close relatives, but not one agreed to be studied (Figure 3).

The new-born was referred for genetic appointments, in order to be studied for the presence of the mutation, but this has not been performed yet.

## Discussion

The present case illustrates the importance of early diagnosis of FPLD2, as well as the challenges faced by medical professionals when prescribing treatment for FPLD2 during gestation, especially considering the limitations imposed on treatment options during pregnancy.

Our patient has demonstrated the typical Dunnigan-variety fat distribution since early adulthood. Before being pregnant, she also experienced two acute and severe episodes of pancreatitis, which were secondary to extremely high triglyceride levels. As occurs in some patients, she was erroneously suspected of having Cushing syndrome, but this hypothesis was excluded.

Despite these clinical manifestations, FPLD2 was only diagnosed during pregnancy. The diagnosis was confirmed by a genetic study. Mutations of the codon 482 of the *LMNA* gene account for the majority of cases reported [20-24,33-35], and, although this residue is traditionally considered a mutational hot spot for FPLD2, other mutations in exon 8 (codon 465, 466, 485 and 486) and in exon 11 (codon 582, 583 and 584) have been recorded [20,33,36].

Lack of fertility and obstetrical complications are frequent with FPLD2. Vantyghem *et al.* reported a prevalence of polycystic ovarian syndrome of more than 50%, of infertility close to 30%, miscarriages of 50%, gestational diabetes of at least 30%, and preeclampsia and foetal death of over 10% [25,32]. Contrary to these previous reports, our patient had no history of polycystic ovarian syndrome, nor any signs of hyperandrogenism, and presented no difficulties in conceiving.

There is no specific treatment for FPLD2, and patients are prone to develop acute pancreatitis, long-term complications of diabetes, liver steatosis and cirrhosis, and also accelerated atherosclerosis.

Currently, the insulin-sensitizing strategy uses lifestyle modifications (diet, physical activity) as a first line of approach. Metformin or glitazones add some benefit when lifestyle modifications alone are not sufficient. However, many patients do not achieve adequate glycaemic control with oral antidiabetic medications, and most require high insulin doses [37-39].

Hypertriglyceridaemia is often resistant to conventional treatment. Fibric acid derivatives (PPARα agonists) can be helpful, as well as high-dose omega − 3 polyunsaturated fatty acids. With some patients, low-dose statins, which also inhibit prelamin A farnesylation, can be added to reduce non-HDL-cholesterol levels [37].

Regarding adipocytokine replacement, the use of recombinant leptin in hypoleptinemic patients with lipodystrophies gives very promising results, with marked improvement in hyperglycaemia, hypertriglyceridemia, and hepatic steatosis [40-43]. Leptin therapy reduces appetite and causes weight loss, which contributes to reducing metabolic complications. In addition to its main effects, leptin also reduces ectopic lipid deposition in the liver and muscle [44]. However, the effects of leptin are less significant in patients with partial lipodystrophies and other laminopathies, than in those with generalised lipodystrophies [41]. In this context, the FDA has recently approved metreleptin (Myalept®) treatment, but only for patients with generalised, inherited and acquired, lipodystrophies [45]. This approval was based on results from an NIH open-label, single-arm study of 48 patients with congenital, or acquired generalized lipodystrophy, who also had diabetes *mellitus*, hypertriglyceridemia, and/or elevated levels of fasting insulin [46].

However, even though leptin replacement seems to be the ideal treatment for metabolic complications in FPLD2 patients, metreleptin is not yet commercially available for these patients. Metreleptin is a pregnancy category C treatment, so it should only be used if the forecast benefit justifies the potential risk for the foetus.

At pregnancy conventional anti-dyslipidemic therapies – such as statins, niacinic acid and some fibric acid derivates – and some oral antidiabetic medications are contraindicated, or at least, not recommended. Adding to these limitations, normal pregnancy is characterised by a 2 to 4-fold increase in plasma triglyceride concentration, and also increased insulin resistance [47]. These changes, which are well tolerated by most women with normal baseline triglyceride levels and no compromised metabolic pathways, do however constitute an increased risk of pancreatitis for women with FPLD2, mainly during a period of life when treatment options are limited.

On account of the risk of pancreatitis, our patient was submitted to an alternative treatment, using highly purified omega-3 fatty acid ethyl esters [48], and a high protein powdered supplement combined with fenofibrate, after discussion of the possible risks involved.

## Conclusion

This case illustrates the importance of a timely diagnosis of FPLD2 in order to avoid severe, and possible life-threatening consequences, such as pancreatitis. The authors intend also to highlight the challenges involved when confronting this condition in pregnant women.

## Consent

Written informed consent was obtained from the patient for publication of this Case Report and any accompanying images. A copy of the written consent is available for review by the Editor-in-Chief of this journal.

## Abbreviations

FPLD2: Familial partial lipodystrophy, Dunnigan variety
*LMNA*: Lamin A/C gene
